# Chemoprevention of Skin Cancer with 1,1-Bis (3′-Indolyl)-1-(Aromatic) Methane Analog through Induction of the Orphan Nuclear Receptor, NR4A2 (Nurr1)

**DOI:** 10.1371/journal.pone.0069519

**Published:** 2013-08-07

**Authors:** Cedar H. A. Boakye, Ravi Doddapaneni, Punit P. Shah, Apurva R. Patel, Chandraiah Godugu, Stephen Safe, Santosh K. Katiyar, Mandip Singh

**Affiliations:** 1 College of Pharmacy and Pharmaceutical Sciences, Florida A&M University, Tallahassee, Florida, United States of America; 2 Department of Veterinary Physiology and Pharmacology, Texas A&M University, College Station, Texas, United States of America; 3 Department of Dermatology, University of Alabama at Birmingham, Birmingham, Alabama, United States of America; UCSF/VA Medical Center, United States of America

## Abstract

**Background:**

The objective of this study was to demonstrate the anti-skin cancer and chemopreventive potential of 1,1-bis(3′-indolyl)-1-(p-chlorophenyl methane) (DIM-D) using an in vitro model.

**Methods:**

In vitro cell cytotoxicity and viability assays were carried out in A431 human epidermoid carcinoma cell line and normal human epidermal keratinocytes (NHEK) respectively by crystal violet staining. Apoptosis induction in A431 cells (DIM-D treated) and NHEK cells pretreated with DIM-D (2 hr) prior to UVB irradiation, were assessed. The accumulation of reactive oxygen species (ROS) in DIM-D pretreated NHEK cells (2 hr) prior to UVB exposure was also determined. Immunocytochemistry and western blot analysis was performed to determine cleaved caspase 3 and DNA damage markers in DIM-D treated A431 cells and in DIM-D pretreated NHEK cells prior to UVB irradiation.

**Results:**

The IC50 values of DIM-D were 68.7±7.3, 48.3±10.1 and 11.5±3.1 μM whilst for Epigallocatechin gallate (EGCG) were 419.1±8.3, 186.1±5.2 and 56.7±3.1 μM for 24, 48 and 72 hr treatments respectively. DIM-D exhibited a significantly (p<0.05) greater induction of DNA fragmentation in A431 cells compared to EGCG with percent cell death of 38.9. In addition, DIM-D induced higher expression in A431 cells compared to EGCG of cleaved caspase 3 (3.0-fold vs. 2.4-fold changes), Nurr1 (2.7-fold vs. 1.7-fold changes) and NFκB (1.3-fold vs. 1.1-fold changes). DIM-D also exhibited chemopreventive activity in UVB-irradiated NHEK cells by significantly (p<0.05) reducing UVB-induced ROS formation and apoptosis compared to EGCG. Additionally, DIM-D induced expression of Nurr1 but reduced expression of 8-OHdG significantly in UVB-irradiated NHEK cells compared to EGCG and UV only.

**Conclusion:**

Our results suggest that DIM-D exhibits Nurr1-dependent transactivation in the induction of apoptosis in A431 cells and it protects NHEK cells against UVB-induced ROS formation and DNA damage.

## Introduction

Skin cancer incidence has been increasing and over 2 million new cases are diagnosed each year in the United States [1]. It has been estimated that one in five Caucasian Americans will develop skin cancer at least once in the course of his/her lifetime [2]. Melanoma is the most severe form of skin cancer and accounts for 5% of all skin cancer cases in America, is responsible for most skin cancer deaths [3] [4], with an impact estimated at $2.36 billion in 2010 [5]. The increasing incidence of skin cancer is expected to continue as the population ages, greater amounts of UV radiation reach the surface of the earth due to depletion of the ozone layer, and continuous use of sun tanning devices [6] [7] [8].

Studies have shown that persistent exposure to sunlight is an important risk factor for development of both nonmelanoma skin cancer (NMSC) and melanoma due to injurious effects of UVB radiation that breaches the epidermal layer of the skin [9] [10]. The initiation and progression of skin carcinogenesis involves a complex cascade of cellular and molecular events ensuing from the initial production of reactive oxygen species (ROS) by UVB radiation [11] [12] [13] and results in keratinocyte DNA damage and mutation including the formation of cyclobutane pyrimidine dimers (CPD). Studies have shown that there is also substantial damage caused to the skin lipids and proteins upon UVB exposure [14] [15].

Many phytochemicals and synthetic analogs have the ability to reverse and/or decrease the onset and progression of skin carcinogenesis and angiogenesis [16] [17]. These phytochemicals are primarily polyphenols, which include but are not limited to silymarin, epigallocatechin 3-gallate (EGCG), curcumin, myricetin, quercetin and hesperitin. EGCG is an abundant polyphenol present in green tea extract and is a potent antioxidant flavonoid that has chemopreventive potential [18]. EGCG can induce cell cycle arrest and apoptosis in hepatoma cells by inducing p53 and Fas/FasL apoptotic pathway respectively [19]. The cytotoxicity of EGCG in vitro requires relatively high concentrations [20] that are not readily achieved in the serum and both oral and topical formulations of EGCG exhibit minimal protection against photoaging and UV-induced inflammatory responses in the skin [21] [22] [23].

3,3′-Diindolylmethane (DIM) (Fig. S1) is a natural product derived from indole-3-carbinol (I3C) which is present in cruciferous vegetables such as brussels sprouts, broccoli and cauliflower. DIM has generated much interest in cancer research because of its low toxicity and cytotoxic effects on cancer cells in vitro and inhibition of tumor growth in vivo [24]. For example, DIM induced expression of cell cycle inhibitors such as p21 and p27 and downregulated-cyclin proteins including cyclin D1 and also decreased expression of survival and antiapoptotic proteins including survivin, bcl-2, bax and induced poly (ADP-Ribose) polymerase (PARP) cleavage, mitochondrial cytochrome c release and procaspase cleavage [25] [26] [27]. A series of novel synthetic 1,1-bis(3′-indolyl)-1-(p-substituted phenyl) methane analogs (C-DIMs), are also potent anticancer agents [25] [28] [29] and their activities are structure-dependent. The p-t-butylphenyl and p-biphenyl derivatives activate peroxisome proliferator-activated receptor γ (PPARγ) whereas the unsubstituted p-phenyl and p-methoxyphenyl analogs activate the orphan receptor NR4A1 (Nurr77/TR3) [30]. Studies in our laboratory have reported a synergistic effect between 1,1-bis(3′-indolyl)-1-(p-biphenyl) methane (DIM-C-pPhC6H5) and Docetaxel in non-small cell lung cancer cells through enhanced induction of cleaved PARP, bax and N-cadherin and inhibition of phospho-Akt, cyclin D1, survivin, NF-kB, Mcl-1 and phospho JNK2 [29] [31].

A member of the nerve growth factor I-B Nurr1 (NR4A2) is another NR4A receptor, which has been implicated, in various hormonal, physiological and pathophysiological processes including cardiovascular, neurological and metabolic diseases, inflammation and oncogenesis. Nurr1 plays a role in brain function and hence has been linked to Alzheimer's disease, Schizophrenia and Parkinson's disease [32]. Nurr1 is highly expressed in Panc1 and Pan28 pancreatic and some human bladder cancer cell lines [28] [33]. In this study, we illustrate the significance of Nurr1 in the chemopreventive potential of 1,1-bis(3′-indolyl)-1-(p-chlorophenyl methane) (DIM-D) in skin cancer using an in vitro UVB induced skin cancer model. DIM-D has shown to induce Nurr1-dependent transactivation and results of our study suggest a possible role for DIM-D/Nurr1 in chemoprevention of skin cancer.

## Materials and Methods

### 1. Materials

p-Substituted C-DIM analogs (DIM-C-pPhCl; DIM-D), (DIM-C-pPhCN; DIM-B) and (DIM-C-pPhBr; DIM-C) were synthesized as described [36]. EGCG was purchased from Selleck Chemicals (Houston, TX, USA). A431 human epidermoid carcinoma cell line and normal human epidermal keratinocytes (NHEK) were acquired from Invitrogen (Grand Island, NY, USA). Phosphate buffered saline (PBS) was purchased from Invitrogen. The NR4A2 (Nurr 1) antibody was obtained from Santa Cruz Biotechnology, Inc. (Santa Cruz, CA). The antibodies directed against 8-hydroxy deguanosine (8-OHdG), CCAAT/-enhancer- binding protein homologous protein (CHOP), nuclear factor kappa-light-chain-enhancer of activated B cells (NF-κB) and cleaved caspase 3 were also obtained from Santa Cruz Biotechnology, Inc. (Santa Cruz, CA).

### 2. Cell lines and cell cultures

A431 cells were maintained in Dulbecco's modified Eagle's medium (DMEM; Sigma Aldrich, St Louis, MO) nutrient mixture supplemented with 10% fetal bovine serum (FBS) from Invitrogen (Grand Island, NY) and antibiotic-antimycotic mixture comprising penicillin (5000 U/mL), streptomycin (0.1 mg/mL), and neomycin (0.2 mg/mL) from Sigma Aldrich (St Louis, MO, USA) at 37°C in the presence of 5% CO_2_ and 95% relative humidity. NHEK cells were maintained in Epilife medium (Invitrogen, Grand Island, NY, USA) and were sustained by either epidermal growth supplement (EDGS) or human keratinocyte growth supplement (HKGS) from Invitrogen (Grand Island, NY) and antibiotic-antimycotic mixture comprising penicillin (5000 U/mL), streptomycin (0.1 mg/mL), and neomycin (0.2 mg/mL) from Sigma Aldrich (St Louis, MO) at 37°C. Both cells lines were sub cultured when approximately 80–90% confluent with 0.25% trypsin-EDTA (Invitrogen; Grand Island, NY). The cells were cultivated on 1.12 cm^2^ 0.4 μm pore polycarbonate membrane inserts in 12 mm×12-transwell permeable support plates (Corning, NY).

### 3. *In vitro* Cell Cytotoxicity and Viability Assays

Cell cytotoxicity and cell viability assays were carried out in A431 and NHEK cells respectively using the conventional crystal violet staining assay. The A431 and NHEK cells were both seeded in 96-well plates (10^4^ cells/well) and incubated overnight in 5% CO_2_ at 37°C. The A431 cells were treated with the solvent control (DMSO) and various concentrations of DIM-B, DIM-C and DIM-D and EGCG, whilst NHEK cells were exposed to UVB radiation (150 J/m^2^) 2 hr following treatment and then incubated for 24 hr in growth media. The A431 cells in drug and media mixture were incubated for 24, 48 and 72 hr. The viable cells were fixed with glutaraldehyde and stained with crystal violet for 15 min at room temperature. The crystal violet was washed; the residue solubilized with sodium hydrogen phosphate and the absorbance was measured with a spectrophotometer at wavelength of 462 nm. Viability of NHEK cells treated with C-DIMs or EGCG were expressed as percentages of the absorbance of control cells, which were regarded as 100% viable and IC50 values were determined by the following formula, [(50– lowest kill)/(highest kill – lowest kill) * (highest conc. – lowest conc.)] + lowest conc.

### 4. TUNEL Assay

A431 cells were treated with 34.4 µM DIM-D (50 percent of IC50 value) and incubated for 24 hr. Cells were then washed with PBS buffer and fixed in 10% formaldehyde on microscopic sides. The ApoTag Red *In Situ* Apoptosis detection kit® (Millipore, Billerica, MA) was used for the detection of apoptosis in accordance with the manufacturer's protocol. Briefly, the fixed cells were incubated in 20 μg/mL proteinase K solution for 15 min at room temp, followed by incubation with equilibration buffer for 10 sec. The cells were washed in PBS and incubated with TdT enzyme at 37°C for 1 hr in a humidified chamber for incorporation of conjugated nucleotides at the 3′-OH ends of DNA. The fixed cells were washed in PBS and incubated with anti-digoxigenin conjugate (Rhodamine Antibody) solution and counterstained with DAPI. The microscopic images of the fixed cells on the slides were visualized with an Olympus BX40 light microscope equipped with a computer-controlled digital camera (DP71, Olympus Center Valley, PA, USA). DNA fragmentation was indicated by Rhodamine positive staining (red). Untreated cells were maintained as control.

### 5. Acridine orange/ethidium bromide staining

Morphological changes in NHEK cell nuclei following exposure to UVB radiation was determined to assess the protective effects of DIM-D and EGCG in the exposed cells. Cells were seeded in 96-well plate (10^4^ cells/well) and treated for 2 hr with different concentrations of solutions of DIM-D and EGCG in DMSO prior to UVB radiation exposure at dose of 150 mJ for 30 sec. Cells were then incubated in growth media for 24 hr and staining was carried out with acridine orange/ethidium bromide (AO/EB) as described [34] and analyzed by fluorescence microscopy. Briefly, the differential uptake of the two dyes was used to ascertain cells viability. After incubation for 24 hr, cells were washed with PBS (2X) and stained with a mixture of acridine orange and ethidium bromide.

### 6. Determination of intracellular reactive oxygen species (ROS)

The fluorescent dye, 2′,7′-dichlorofluorescein diacetate (DCF-DA) was used to assess the accumulation of ROS in NHEK cells after UVB exposure. Cells were seeded in a 24-well plate at a density of 0.05×10^6^ cells/well, treated with different concentrations of DIM-D and EGCG in DMSO for 2 hr, aspirated a thin film of PBS was added and cells were then exposed to UVB radiation dose of 150 mJ for 30 sec. Cells were then incubated for 24 hr in growth media only. DCF-DA solution (10 μM) was added to the cells (0.05×10^6^/mL) and the mixture incubated at 37°C for 1 hr in the dark. Cells were washed with PBS (2X) and the fluorescence intensity of the cells was determined by fluorescence microscopy.

### 7. Immunocytochemical analysis

Both A431 and NHEK cells were treated with DIM-D and EGCG (34.4 μM and 210.0 μM respectively) and incubated for 24 hr. Cells were then fixed in 10% formaldehyde and cytospun into microscopic slides. The analysis was carried out following the protocol specified in the SignalStain^TM^ IHC kit (Cell Signaling, Beverly, MA). Fixed cells were hydrated with varying concentrations of alcohol and PBS (3X), incubated with the primary antibodies against cleaved caspase 3, Nurr1 and 8-OHdG overnight at 4°C and detected by HRP-conjugated secondary antibody. Cells were stained with Nova Red stain and counterstained with hematoxylin. Microscopic analysis of the fixed cells was carried out with an Olympus BX40 light microscope equipped with computer-controlled digital camera (DP71, Olympus Center Valley, PA, USA).

### 8. Western blot analysis

Proteins were collected from both A431 and NHEK cells as described [33]. Briefly, after 24 hr treatment with DIM-D (17.2 μM) and EGCG (104.8 μM), cells were treated with RIPA buffer (50 nM Tris-HCl, pH 8.0, with 150 mM sodium chloride, 1.0% Igepal CA-630 (NP-40), 0.5% sodium deoxychlorate, and 0.1% sodium dodecyl sulfate) with protease inhibitor (500 mM phenylmethylsulfonyl fluoride). Protein concentrations were determined according to BCA protein assay reagent protocol (PIERCE, Rockford, IL) and the standard plot was generated by using bovine serum albumin, 50 μg of supernatant protein from the control group and all the different treatment samples were denatured by boiling at 100°C for 5 min in SDS sample buffer and were subsequently electrophoresed in 10% SDS-PAGE gel, transferred into nitrocellulose membranes, blocked with 5% skim milk in tris-buffered saline with tween 20 (10 mM tris-HCl (pH 7.6), 150 mM NaCl, and 0.5% Tween), and probed with antibodies against Nurr1 (1∶400), NFκB (1∶500) and cleaved caspase 3 (1∶1000) for A431 cells. Proteins were detected with HRP conjugated secondary antibodies using SuperSignal West pico chemiluminescent solution (PIERCE, Rockford, IL). Quantification and analysis of the results were carried out with Image J software (v1.33u, NIH, USA). Results were expressed as percentage ratios of protein expression to β-actin (set to 100%).

### 9. Statistical analysis

Results are expressed as the mean ± S.D for at least three replicates and comparison between multiple groups was established by a one-way analysis of variance (ANOVA) and between two groups by student's t test analysis. A p value <0.05 was considered significant.

## Results

### 1. Anticancer activity of DIM analogues

#### 1.1 Effect of DIM analogues on A431 skin cancer cells

**Figure 1:**
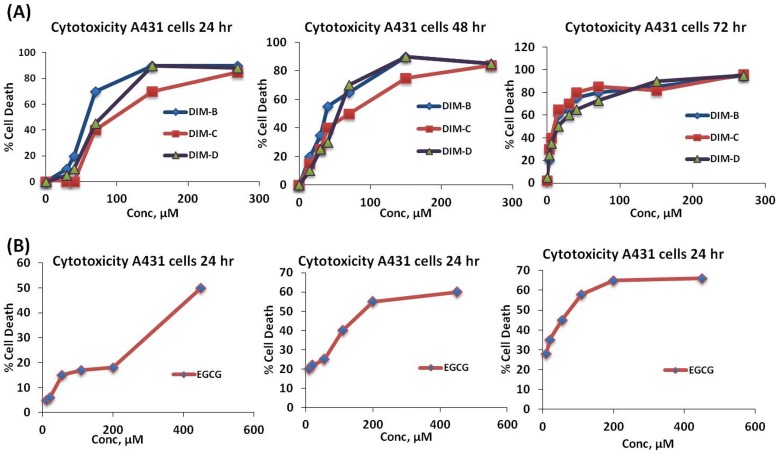
Cytotoxicity profile of DIM analogues and EGCG at different concentrations in A431 cells. (**A**) Cytotoxicity profile (Plot of % cell death vs concentration of drug at A) 24 hr, B) 48 hr and C) 72 hr where, DIM-B  =  DIM-C-pPhCN, DIM-C  = DIM-C-pPhBr and DIM-D  =  DIM-C-pPhCl). Data represent mean ± SD. (**B**) Cytotoxicity profile (Plot of % cell death vs concentration of drug) at A) 24 hr, B) 48 hr, C) 72 hr for EGCG in A431 cells.

A431 cells were treated with three different DIM analogues, which differ structurally at the p-phenyl position (Fig. S1). A431 cells were treated with DIM-B for 24, 48 and 72 hr and IC50 values for cytotoxicity were 56.8±10.7, 30.8±6.6 and 7.2±1.2 μM respectively (Fig. 1A). The IC50 values for DIM-C after treatment for 24, 48 and 72 hr were 78.3±16, 50.2±12.2 and 7.2±1.5 μM respectively and the corresponding IC50 values for DIM-D were 68.7±27.3, 48.3±10.1and 11.5±3.1 μM after treatment for 24, 48 and 72 hr. In contrast, IC50 values for EGCG after treatment for 24, 48 and 72 hr were 419.1±8.3, 186.1±5.2 and 56.7±3.1 μM respectively (Fig. 1B), which were significantly higher than observed for C-DIMs (Table 1). All three DIM derivatives showed more potency than EGCG with little variations between their potencies. DIM-D was however selected for further studies as a model drug because it exhibited more photostability than DIM-B and DIM-C.

**Table 1:** IC50 values of DIM-B (DIM-C-pPhCN), DIM-C (DIM-C-pPhBr) and DIM-D (DIM-C-pPhCl) and ECGC at 24, 48 and 72 hr. Data represent mean ± SD (n = 3).

Time (hr)	IC50 values µM			
	DIM-B	DIM-C	DIM-D	Epigallocatechin gallate (EGCG)
24	56.8±10.7	78.3±16.0	68.7±7.3	419.1±8.3
48	30.8±6.6	50.2±12.2	48.3±10.1	186.1±5.2
72	7.2±1.2	7.2±1.5	11.5±3.1	56.7±3.1

#### 1.2 Apoptotic activity of DIM-D against A431 cells

**Figure 2:**
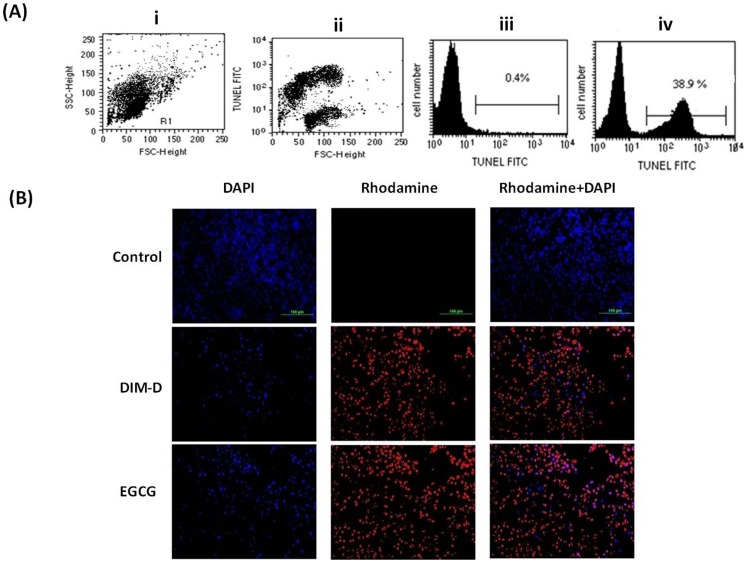
Determination of apoptosis in A431 cells after treatment with DIM-D and EGCG by TUNEL assay. (**A**) Detection of apoptotic cells by the TUNEL method using flow cytometric analysis. A431 cells were stained for apoptotic cells with FITC by the TUNEL method before and after 24 hr treatment. (i) Dot plot depicting FSC versus SSC profile of cells after treatment. (ii) Dot plot depicting profile of FITC fluorescence versus FSC for cells after treatment. Most of the TUNEL-stained apoptotic cells are slightly smaller than unstained live cells. (iii) Histogram depicting cells before treatment in the gate R1. Very few apoptotic cells are detected. (iv) Histogram depicting cells after treatment with DIM-D (DIM-C-pPhCl) in the gate R1. Apoptotic cells (brightly stained by TUNEL) are detected. (**B**) Detection of apoptotic cells by the TUNEL method using microscopic analysis. A431 cells were stained for apoptotic cells with Rhodamine by the TUNEL method for 24-hr treatment. Cells were counterstained with Hoechst dye (DAPI).

The effects of DIM-D on apoptosis in A431 cells were examined by the TUNEL method using flow cytometry. A431 cells treated with DIM-D (34.4 µM) for 24 hr significantly induced cell death (38.9%) compared to DMSO control (Fig. 2A). We also investigated the effects of DIM-D and EGCG on DNA fragmentation of A431 cells using TUNEL method by microscopic analysis (Fig. 2B). In control cells treated with PBS, minimal DNA fragmentation was observed whereas DIM-D significantly increased DNA fragmentation as evidenced by increased red fluorescence. Similarly, EGCG (104.8 μM) also increased DNA fragmentation but comparatively less when compared with DIM-D (Fig. 2B).

#### 1.3 Effect of DIM-D on apoptotic proteins and Nurr1

**Figure 3:**
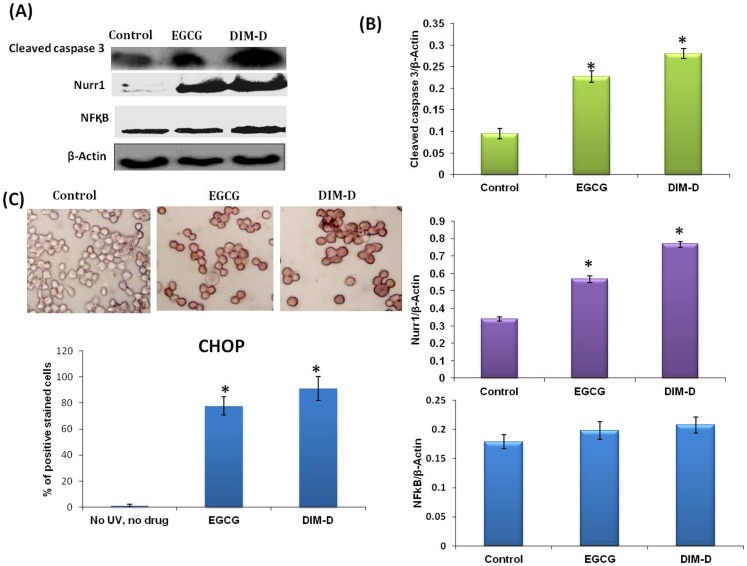
Determination of protein expression levels by western blot and immunocytochemistry. (**A**) Expression of cleaved caspase 3, Nurr1 and NFκB in comparison to the control β-actin in Dim-D and EGCG treated A431 cells by western blot. Untreated cells were maintained as control. (**B**) Western blot analysis of expression of different proteins increased significantly in DIM-D treated cells. (**C**) ICC peroxidase staining of A431 cells treated with DIM-D and EGCG, respectively for the pro-apoptotic protein, CHOP. Brown staining was considered positive result. Untreated cells were maintained as control. Data are calculated from triplicate experiments and presented as mean, and error bars refer to SD, *P<0.05, **P<0.01 compared with control.

A431 cells were treated with 34.4 µM DIM-D and 104.8 μM EGCG for 24 hr and whole cell lysates were analyzed by western blots (Fig. 3A). DIM-D significantly induced expression of cleaved (activated) caspase-3 and Nurr1 proteins and similar results were observed for EGCG. Quantitation of these results (Fig. 3B) showed that DIM-D was more potent that EGCG as an inducer of cleaved caspase-3 (3.0 fold vs 2.4 fold) and Nurr1 (2.7 fold vs 1.7 fold) whereas effects on NFκB (p65) were minimal (1.3 fold vs 1.1 fold). Expression of cleaved caspase-3 and Nurr1 was significantly induced by DIM-D and EGCG (p<0.05). DIM-D (34.4 µM) and EGCG (104.8 μM) also induced CHOP expression as indicated by immunostaining of A431 cells after treatment for 24 hr (Fig. 3C). Quantitation of these results showed significant increase of CHOP positively stained cells (79%) in DIM-D treated cells as compared to 67% of cells positively stained after EGCG treatment. These results suggest that the anti-skin carcinogenic potency of DIM-D is greater than EGCG under identical conditions.

### 2. Chemopreventive activity of DIM-D on normal human epidermal keratinocyte (NHEK) cells

#### 2.1 Cytotoxic effect of DIM-D on NHEK cells

**Figure 4:**
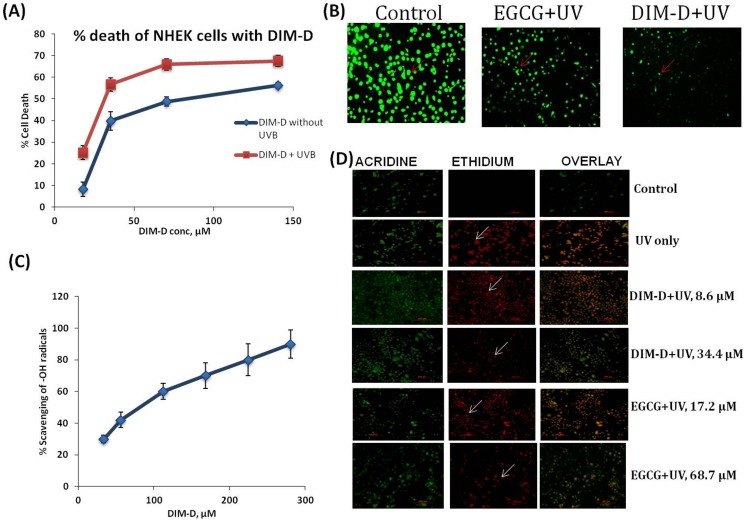
Effect of cytotoxicity, antioxidant potential and apoptosis in NHEK cells treated by DIM-D for 24 hr. (**A**) Line graph of cytotoxicity profile (Plot of % cell death vs concentration of drug) of NHEK cells treated with DIM-D (DIM-C-pPhCl) at various concentrations with and without UVB radiation. Data represent mean ± SD. (**B**) Production of Reactive oxygen species (ROS) with DIM-D and EGCG treatment prior to UVB exposure. Each value is expressed as the mean± standard deviation. (**C**) Hydroxyl radical scavenging in an in vitro cell-free system treatment by DIM-D. The data is expressed in terms of percentage of control in which the cell-free system was not treated with DIM-D. (**D**) Apoptosis determination in NHEK cells treated with DIM-D (DIM-C-pPhCl) and EGCG at different concentrations.

In order to investigate the effects of DIM-D against normal skin cells, NHEK cells were treated with various concentrations of DIM-D (15 to 140 µM) for 24 hr and results indicated that DIM-D was minimally toxic to NHEK cells (Fig. 4A). At the maximum concentration of 140 µM, percentage cell death was 56.2±1.3 whilst at the IC50 concentration (68.7±7.3 µM) observed in A431 cells, NHEK cell death was reduced to 48.7±2.1%. Hence, concentration of DIM-D 68.7±7.3 µM was employed for all subsequent investigations. On the other hand, exposure of the pretreated NHEK cells to UV radiation, reduced cell viability further. This was however not significant with increase in percent cell death to 67.5±2.6 and 65.9±3.1 for 140 µM and 68.7 µM respectively (Fig. 4A).

#### 2.2 Anti-oxidant potential and apoptotic activity of DIM-D on NHEK cells

The antioxidant activity of DIM-D and protection against UV-induced ROS was also investigated in NHEK cells treated with 34.4 µM DIM-D and 209.6 µM EGCG for 24 hr. The UVB-induced ROS levels in NHEK cells pretreated with DIM-D were significantly reduced compared to EGCG; this demonstrates that DIM-D has more potent anti-oxidant activity than EGCG (Fig. 4B). In addition, the ability of DIM-D to scavenge hydroxyl (-OH) radicals in a cell-free in vitro system was utilized to confirm the enhanced anti-oxidant potential of DIM-D compared to EGCG. The results in Figure 4C revealed that at concentrations of 34.4 µM to 280 µM, DIM-D was able to quench approximately 30% to 90% of the hydroxyl radicals.

Next, apoptotic activity was analyzed in NHEK cells, which were pretreated with EGCG (17.2 µM and 68.7 µM) and DIM-D (8.6 and 34.4 µM) prior to UVB exposure. For efficient comparison between EGCG and DIM-D with respect to protection against apoptosis, the IC50 and one-fourth the IC50 concentrations of DIM-D were employed for EGCG pretreatment of cells whilst half of the respective concentrations were employed for DIM-D pretreatment. The acridine orange/ethidium bromide double staining was utilized for the apoptotic assay because the acridine orange dye has ability to stain the nuclei of viable cells green whilst the ethidium dye, an intercalating agent permeated apoptotic or necrotic cells to stain their nuclei orange. We demonstrated that in cells, which were treated with DIM-D, there was a significant reduction apoptotic cell death (red staining) compared to cells treated with EGCG (Fig. 4D). No apoptotic cell death was observed in the control cells, which were treated with either PBS or vehicle only. DIM-D treatment caused significant decrease in induction of apoptosis by UVB irradiation in NHEK cells. EGCG, however, showed minimal protection of the cells against apoptosis. The cells pretreated with EGCG prior to UVB irradiation were markedly stained red by ethidium bromide. Overall, these findings suggest that although there was reduction in cell viability in DIM-D+UV treated cells compared to DIM-D only treated cells (as shown in fig. 4A); DIM-D treatment relatively prevents the induction of apoptosis by UVB radiation and hence enhances cell viability more significantly than EGCG.

#### 2.3 Effect of DIM-D on UVB-induced-oxidative stress

**Figure 5:**
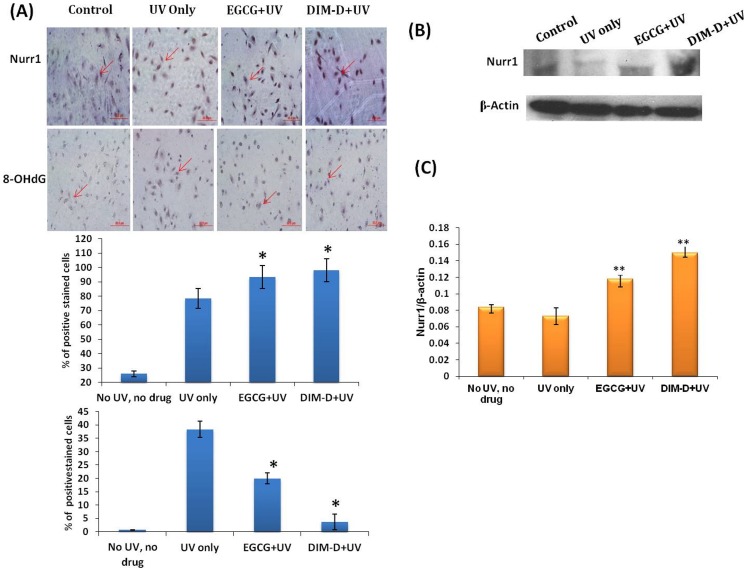
Expression of Nurr1 and 8-OHdG in NHEK cells by immunocytochemistry and further confirmation by western blot. (**A**) Immunocytochemical peroxidase staining of Nurr1 and 8-OHdG in NHEK cells treated with DIM-D and EGCG. Presence of brown stain indicated positive staining for the primary antibody. (**B**)**&**(**C**) Western blot analysis of expression of Nurr1 in comparison to the control β-actin in Dim-D and EGCG treated NHEK cells. Untreated cells were maintained as control. Data are calculated from triplicate experiments and presented as mean± SD, *P<0.05, **P<0.01 compared with control.

To further investigate whether DIM-D inhibits UV radiation-induced oxidative stress and inflammation through enhancement of Nurr1 in UV-exposed skin cells, we examined the expression of Nurr1 and 8-OHdG in NHEK cells (Fig. 5). After UVB irradiation, cells were treated with DIM-D (34.4 µM) and EGCG (209.6 µM) for 24 hr and formation of 8-OHdG was determined by immunostaining (Fig. 5A). UVB significantly increased 8-OHdG but both DIM-D and EGCG significantly decreased the UVB-induced response thus confirming the antioxidant activity of both compounds. Their relative potency was evident by immunostaining. Only 3.6% of cells were positively stained in DIM-D treatment as compared to EGCG treated cells (20% cells).

Immunocytochemical analysis revealed that, the Nurr1 expression was significantly stronger as compared to EGCG and UV only (Fig. 5A). Quantitation of these results showed that approximately 83% of cells were positively stained for Nurr1 in DIM-D treated cells as compared to EGCG treated cells (66%). These results clearly demonstrated that DIM-D was more potent that EGCG as an inducer of Nurr1. Further these results were confirmed by western blot analysis. For this purpose, NHEK cells were treated with DIM-D (34.4 µM) and EGCG (209.6 µM) for 24 hr and whole cell lysates were analyzed (Fig. 5B). NHEK cells treated with DIM-D showed Nurr1 expression was increased significantly more in DIM-D treated cells (1.8 fold) as compared to EGCG (1.4 fold) (Fig. 5C).

## Discussion

Poor clinical outcome of the current treatment avenues has prompted the need for the development of new therapeutic strategies for treatment of skin cancer. There is an urgent need to identify molecular targets using dietary compounds, which have both therapeutic as well as chemopreventive activity. Nurr1 is an orphan nuclear receptor and preliminary reports suggest a role for Nurr1 in rheumatoid arthritis and cancer through modulation of apoptosis. Further it has also been demonstrated that DIM analogues activates the orphan nuclear receptor Nurr1 and inhibits bladder cancer growth [28]. However there are no reports on the chemopreventive potential of DIM-D activated Nurr1 in skin cancer. Therefore, in the first part of present study we focused on anticancer or therapeutic activity of DIM-D using A431 skin cancer cells. In the second part of our study we investigated the chemopreventive activity of DIM-D through Nurr1 mediated signaling using NHEK.

In first part of our study, we assessed the cytotoxic effect of DIM-D on A431 skin cells. It is important to point out that DIM-D showed cytotoxic effect on these cells and its cytotoxic effect was greater than EGCG. Similar to EGCG, previous studies have demonstrated that DIM inhibits growth of cancer cells derived from tumors of the prostate, breast, colon, cervix and pancreas [34]. The role of apoptosis was further investigated by determining expression of apoptotic protein such as cleaved caspase-3 in A431 cells. The western blot analysis showed significant increase in expression of caspase-3 following DIM-D treatment in A431 cells. We also studied DNA fragmentation in A431 cells after DIM-D treatment because DNA fragmentation is a hallmark of apoptosis, which commits cells to die. DNA fragmentation was highly induced by DIM-D compared to EGCG, thus confirming that apoptosis is an important pathway associated with the anticancer activity of these compounds. This was well correlated with our previous study of enhancement of anticancer activity by a DIM compound in human non-small cell lung cancer cells [29]. Previous studies have shown that DIM-D activates endoplasmic reticulum stress in pancreatic and ovarian cancer cells [35] [36]. DIM-D induced expression of endoplasmic reticulum stress protein GRP78 through enhanced expression of CHOP and this was accompanied by inhibition of tumor growth [37]. Similarly, our immunocytochemical studies showed that DIM-D increased the expression of CHOP in A431 cells after treatment for 24 hr. These results demonstrate that DIM-D exerts its anti-cancer effects through targeting multiple molecular targets associated with cell survival and apoptosis.

Overexpression of Nurr1 decreases inflammatory mediators, scavenger receptor expression and lowers LDL accumulation in macrophages [38] [39]. In our study, expression of cleaved caspase-3 was increased in A431 cells after DIM-D treatment. The repression of inflammatory markers including NFκB provide protection to normal cells from the damaging effects of UVB irradiation [40] whilst on the other hand, their stimulation in cancer cells can induce stress and subsequently, apoptosis. This is in complete concordance with our study where we have shown the pronounced upregulation of NFκB in A431 cancer cells treated with DIM-D and to a lesser extent, in EGCG treated cells, all compared to control. NFκB regulates the expression of genes involved in many processes that play a key role in the development and progression of cancer such as proliferation, migration and apoptosis.

In second part of our study, we assessed the chemopreventive effect of DIM-D in NHEK. For this purpose, cells were exposed to UVB with and without treatment with DIM-D to examine the cytotoxic effect. It is important to point out that DIM-D relatively did not show cytotoxic effect on normal cells but after exposing these cells to UVB, the viability of the cells were reduced further. In spite of this observation, the acridine orange/ethidium bromide double staining revealed the relative cytoprotective effect of DIM-D in the NHEK cells compared to EGCG. Hence, though percentage cell death increased with DIM-D+ UV treatment, there was protection to an extent against induction of apoptosis in these cells. Another explanation of this observation is the fact that this effect of DIM-D on UVB-irradiated cells protects the photodamaged cells from further proliferation, which may be mutated or malignant. Thus, it shows the chemopreventive activity of DIM-D. Excessive exposure of the skin to solar UV- radiation is one of the major etiologic factors for the development of skin cancer.

Hence, after exposing NHEK cells to UVB radiation, we investigated the antioxidant potential of DIM-D. Here we also used EGCG because of its known anticarcinogenic and antioxidant activities [41]. The present study demonstrates that DIM-D and EGCG reduced ROS levels in UVB irradiated NHEK cells. The induction of oxidative stress also overwhelms the antioxidant defense ability of the cutaneous system and leads to the onset of several disease states including skin cancer or photocarcinogenesis and photoaging [42]. It was reported that dietary grape seed proanthocyanidins also inhibit UVB-induced photocarcinogenesis in mice by reducing the levels of UVB-induced oxidative stress [43]. In our study, the hydroxyl radical scavenging activity of DIM-D in an in vitro cell-free system was pronounced suggesting that the DIM-D will protect normal skin against UVB induced oxidative stress, which leads to photocarcinogenesis. Our results were consistent with Wiseman et al [44] who demonstrated that green tea extract effectively scavenged superoxide free radicals, hydroxyl radicals and prevented Cu-mediated LDL oxidation. The anti-oxidant effect of DIM-D was however superior to EGCG because treatment with DIM-D scavenged more hydroxyl radicals as compared with EGCG.

To investigate the possible involvement of apoptosis in the chemopreventive effects of DIM-D, we examined apoptosis in UVB irradiated NHEK cells after DIM-D treatment. Our results demonstrated that in UVB irradiated NHEK cells; apoptosis was low as compared to EGCG showing its chemopreventive activity was superior to that of EGCG. EGCG has been reported to have anticancer activity in various cancers and demonstrated that EGCG inhibited the growth of squamous carcinoma cell's via S and G(2)/M phase arrest [45]. Although the molecular mechanisms of DIM-D action are not yet clearly understood, it appears to have potential as a therapeutic agent. Recently Katiyar et al [46] also demonstrated that treatment of NHEK with silymarin inhibits UVB-induced apoptosis of keratinocytes, and in this process UVB-induced DNA damage was significantly reduced or repaired after silymarin treatment.

Nurr1 is known to play important role in regulating inflammatory disease. The inflammatory response contributes to the pathogenesis of the sunburn reaction, photocarcinogenesis, photoaging of the skin and UV-induced immune suppression [47]. Nurr1 is induced by atherogenic stimuli in macrophages and smooth muscle cells and is found in atherosclerotic plaques [42]. In the current study, western blot and immunocytochemical analysis showed that in UVB irradiated NHEK cells, expression of Nurr1 was significantly induced in DIM-D and EGCG treated cells but expression was higher in DIM-D treated cells. According to Bensinger&Tontonoz, examination of isolated microglia and astrocytes demonstrated that Nurr1 potently influences inflammatory gene expression in these cells [48]. Measurement of the levels of 8-OHdG is used as a biomarker of oxidative stress [49]. The molecular marker 8-OHdG expression was decreased after DIM-D treatment, showing its superior activity of reducing UVB-induced oxidative stress.

Nurr1 mediated signaling by DIM-D decreased UVB-induced skin damage and may play vital role in complex pathways of cell survival and apoptosis of cancer cells. Previous studies suggested that DIM-D activates Nurr1 in human bladder cancer cells and inhibits bladder tumor growth [28] [3]–[4]. Therefore, it is expected that Nurr1 mediated signaling by DIM-D would inhibit initiation and promotion stages of UVB-induced photocarcinogenesis in skin. Like silymarin, DIM-D may act as chemotherapeutic agents by sensitizing tumors. In addition to its chemopreventive effects, silymarin exhibits antitumor activity against human tumors (e.g., prostate and ovary) in rodents [50]. Likewise, our study also suggests that DIM-D shows anticancer and chemopreventive activities and more importantly this activity was superior to well known natural anticancer compound EGCG. Therefore, the mechanism involved in chemoprevention of DIM-D through Nurr1 mediated signaling is novel and in future, will be confirmed further by using Nurr1 deficient mice. The overall mechanism of DIM-D suggested in this manuscript is the fact that DIM-D has potential to induce inflammation, oxidative stress and apoptosis which is evident in the induction of NFκB, CHOP and cleaved caspase 3, in the skin cancer cells to result in death of the cancer cells through the induction and mediation by the orphan nuclear receptor, Nurr1. Our study also suggests that DIM-D is a potential novel agent for developing chemopreventive strategies against UVB induced skin cancer because of its significant reduction in DNA damage and mutation and apoptosis of cells after UV irradiation.

In summary, data from this study suggest that DIM-D may be efficacious in treating skin cancer by acting as a potent and specific stimulator of the Nurr1-mediated apoptosis in skin cancer cells. Further studies are required to evaluate the chemopreventive effects of DIM-D on skin cancer development using in vivo animal models.

## Supporting Information

Figure S1 Structure of DIM analogues (DIM-C, DIM-B and DIM-D).
(JPG)
