# Supplementary figures and images for: Chemoprevention of Skin Cancer with 1,1-Bis (3′-Indolyl)-1-(Aromatic) Methane Analog through Induction of the Orphan Nuclear Receptor, NR4A2 (Nurr1)
Source: PLoS One. 2013 Aug 7;8(8):e69519. doi: 10.1371/journal.pone.0069519 (PMC3737220; doi:10.1371/journal.pone.0069519)

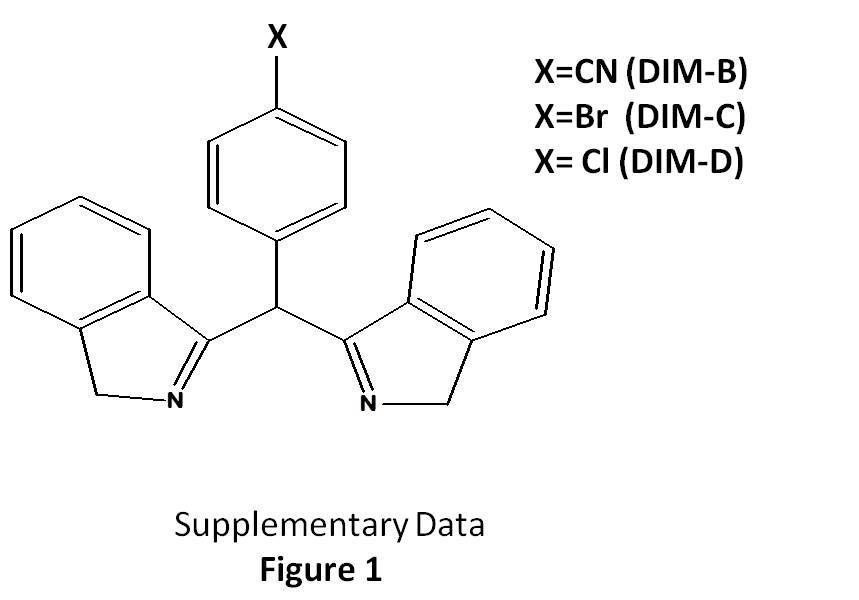

Supplement: Figure S1 — Structure of DIM analogues (DIM-C, DIM-B and DIM-D). (JPG) [file pone.0069519.s001.jpg]
